# Host cell and expression engineering for development of an *E. coli *ketoreductase catalyst: Enhancement of formate dehydrogenase activity for regeneration of NADH

**DOI:** 10.1186/1475-2859-11-7

**Published:** 2012-01-11

**Authors:** Katharina Mädje, Katharina Schmölzer, Bernd Nidetzky, Regina Kratzer

**Affiliations:** 1 Institute of Biotechnology and Biochemical Engineering, Graz University of Technology (TUG), Petersgasse 12/1, A-8010 Graz, Austria

## Abstract

**Background:**

Enzymatic NADH or NADPH-dependent reduction is a widely applied approach for the synthesis of optically active organic compounds. The overall biocatalytic conversion usually involves *in situ *regeneration of the expensive NAD(P)H. Oxidation of formate to carbon dioxide, catalyzed by formate dehydrogenase (EC 1.2.1.2; FDH), presents an almost ideal process solution for coenzyme regeneration that has been well established for NADH. Because isolated FDH is relatively unstable under a range of process conditions, whole cells often constitute the preferred form of the biocatalyst, combining the advantage of enzyme protection in the cellular environment with ease of enzyme production. However, the most prominent FDH used in biotransformations, the enzyme from the yeast *Candida boidinii*, is usually expressed in limiting amounts of activity in the prime host for whole cell biocatalysis, *Escherichia coli*. We therefore performed expression engineering with the aim of enhancing FDH activity in an *E. coli *ketoreductase catalyst. The benefit resulting from improved NADH regeneration capacity is demonstrated in two transformations of technological relevance: xylose conversion into xylitol, and synthesis of (*S*)-1-(2-chlorophenyl)ethanol from *o*-chloroacetophenone.

**Results:**

As compared to individual expression of *C. boidinii *FDH in *E. coli *BL21 (DE3) that gave an intracellular enzyme activity of 400 units/g_CDW_, co-expression of the FDH with the ketoreductase (*Candida tenuis *xylose reductase; XR) resulted in a substantial decline in FDH activity. The remaining FDH activity of only 85 U/g_CDW _was strongly limiting the overall catalytic activity of the whole cell system. Combined effects from increase in FDH gene copy number, supply of rare tRNAs in a Rosetta strain of *E. coli*, dampened expression of the ketoreductase, and induction at low temperature (18°C) brought up the FDH activity threefold to a level of 250 U/g_CDW _while reducing the XR activity by just 19% (1140 U/g_CDW_). The *E. coli *whole-cell catalyst optimized for intracellular FDH activity showed improved performance in the synthesis of (*S*)-1-(2-chlorophenyl)ethanol, reflected in a substantial, up to 5-fold enhancement of productivity (0.37 g/g_CDW_) and yield (95% based on 100 mM ketone used) as compared to the reference catalyst. For xylitol production, the benefit of enhanced FDH expression was observed on productivity only after elimination of the mass transfer resistance caused by the cell membrane.

**Conclusions:**

Expression engineering of *C. boidinii *FDH is an important strategy to optimize *E. coli *whole-cell reductase catalysts that employ intracellular formate oxidation for regeneration of NADH. Increased FDH-activity was reflected by higher reduction yields of D-xylose and *o*-chloroacetophenone conversions provided that mass transfer limitations were overcome.

## Background

Enzymatic reductions are widely used for the preparation of single-isomer alcohols, amino acids and other fine chemicals. Reductases and dehydrogenases applied in biocatalysis usually utilize NADP(H) or NAD(H) as cofactors. The *in situ *recycling of the high priced and labile coenzymes remains a dictate of economic considerations. *Candida boidinii *formate dehydrogenase (*Cb*FDH) has been employed as a workhorse for NADH-regeneration for decades and is used in one of the biggest processes of chiral synthesis, the production of *tert*-L-leucine [1,2]. The irreversible oxidation of formate to carbon dioxide pulls coupled reactions to complete conversion and therefore turned out as an optimal solution from a process point of view. Moreover principles of green chemistry are met by the low molecular weight co-substrate formate whose oxidation product CO_2 _is innocuous and evaporates from the reaction mixture. The general sensitivity of the known FDHs to organic solvents, however, prevents use of FDH in conversion of substrates with low water solubility. Recombinant *E. coli *strains co-expressing FDH and a reductase provide whole-cell biocatalysts that offer the advantage of protecting the enzyme against adverse medium effects in the cellular environment [3,4]. Whole cell bioreduction systems allow furthermore the production of the required activities for carbonyl reduction and coenzyme recycling by single bioreactor cultivation without further enzyme isolation. Efficiencies of whole cell reductions depend therefore on intracellular activities of the reductase and the cofactor recycling dehydrogenase. Previously reported *E. coli *whole cell catalysts based on *Cb*FDH were limited by low FDH activity in *E. coli*, especially under conditions of high reductase co-expression. *Cb*FDH is therefore rarely used in whole cell systems [3-6] whereas many papers have been published on the corresponding conversions catalyzed by free enzymes.

The aim of the present work was to improve a previously developed whole cell biocatalyst based on *Candida tenuis *xylose reductase (*Ct*XR) by boosting the intracellular *Cb*FDH-level [3,4]. Strains differing in coenzyme recycling capacity were constructed and tested for productivities and yields in the production of two industrially relevant chemicals, xylitol and (*S*)-1-(2-chlorophenyl)ethanol. The exceptional substrate promiscuity of *Ct*XR allowed the application of two reporter systems differing to a great extent in reactivity, polarity and toxicity. We thereby could show that limitations in whole cell reductions are strongly case-specific and substrate-dependent. Reduction yields obtained with whole cells as compared to cell free extracts permitted investigation of the role of mass transfer in the case of hydrophilic xylose whereas information on enzyme protection was provided when working with toxic *o*-chloroacetophenone.

## Results and Discussion

*E. coli *has been known to accumulate recombinant protein to a level of up to 50% of the total cell protein [7]. Because of this very high protein production capacity, *E. coli *is the most widely used host organism in whole-cell biotransformations. However, the design of *E. coli *whole cell catalysts for ketone reduction must consider the complication that the activity of the ketoreductase should be balanced to the activity of the dehydrogenase applied for coenzyme regeneration. Finely tuned co-expression of the genes of interest is therefore required and standard solutions are currently not available to achieve this goal. We have shown in prior work that in *E. coli *cells expressing *Cb*FDH next to *Ct*XR, the activity of *Cb*FDH was 4.8-fold lower in comparison to the activity produced under *Cb*FDH-only expression conditions [3,4]. We have carried out this study with the aim of eliminating through the application of different strategies of cell and expression engineering the bottleneck of NADH regeneration in *E. coli *whole cells containing *Cb*FDH and *Ct*XR. Gene copy number, promoter strength, codon usage, mRNA stability and translation velocity were factors taken into consideration.

### Optimization of *Cb*FDH production under single-gene expression conditions

When expressed from the previously described plasmid vector pBTac1, production of *Cb*FDH in *E. coli *JM109 resulted in an activity of 408 U/g_CDW _(Table 1, JM109_FDH). Based on a specific activity of 4.4 U/mg for purified *Cb*FDH, it was estimated from the protein content in the *E. coli *extract that *Cb*FDH had accumulated to about 18% of the total soluble protein. These numbers [8] serve as reference in the evaluation of the new *E. coli *constructed in this work.

**Table 1:** FDH activities measured in the crude cell extracts of single- and co-expression strains at two induction temperatures.

*E coli *strain	*Cb*FDH (U/g_CDW_) at 25°C	*Cb*FDH (U/g_CDW_) at 18°C
JM109_FDH	408 (reference, [8])	n.d.

BL21_FDH	402 ± 38^1^	450

Star_FDH	458	551

Rosetta_FDH	445	614

BL21_XR_FDH	85 ± 12^2^	110 ± 20^2^

BL21_XR_2FDH	146 ± 30^1^	186 ± 30^2^

Rosetta_XR_FDH	100 ± 18^1^	138 ± 23^2^

Rosetta_XR_2FDH	202 ± 21^1^	251 ± 50^2^

^1^n ≥ 3; ^2^n ≥ 7; n.d. not determined.

#### Effects of gene copy number and promoter strength

The vector pBTac1 is used for protein expression of medium strength in *E. coli*. Target genes are cloned under control of the *tac *promoter/*lac *repressor system into the pBR322-derived vector. The moderate to high strength of the *tac *promoter is compromised by a low gene dosage of 15 -20 copies per cell [7]. The used strain JM109 is designed as cloning strain and generally not used as high level protein expression strain. *E coli *expression strains are deficient in two key proteases whereas JM109 lacks only one of these protease activities [9,10]. An increase in copy number and enhancement of promoter strength were considered as potentially useful strategies for improved *Cb*FDH production. We chose pRSF-1b, an expression system for protein expression of up to 50% of the total *E. coli *protein due to a copy number of > 100 and the strong T7*lac *promoter. The constructed pRSF-1b_FDH was transformed into BL21 (DE3), a host providing the T7 polymerase in *trans *and lacking key proteases [9,10]. Recombinant enzyme production in *E. coli *BL21 (DE3) gave an activity (402 U/g_CDW_) essentially identical to that of the reference activity (Table 1, JM109_FDH). These results suggested that the amount of transcript formed is not the main limitation in the expression of *Cb*FDH in *E. coli*. In prokaryotic cells, ribosomes translate mRNAs that are still being transcribed from DNA and thereby protect mRNAs from degradation. Fast mRNA synthesis by the T7 system, however, uncouples transcription from translation in *E. coli *and exposed mRNA is subject to enzymatic degradation [11]. We therefore studied the effect of improved mRNA stability.

#### Effect of enhanced mRNA stability

The average half-life of mRNA in *E. coli *at 37°C ranges from seconds to maximally 20 min, and the expression rate depends directly on the inherent stability of the mRNA [12]. Increased mRNA lifetime might thus translate directly into enhanced *Cb*FDH production. We therefore expressed pRSF-1b_FDH in the RNaseE deficient *E. coli *strain BL21 star (DE3). RNaseE (*rne *131) has been reported to be a major source of mRNA degradation in *E. coli *[9]. Functional expression of *Cb*FDH was improved by about 14% in *E. coli *BL21 star (DE3) as compared to the reference (Table 1, JM109_FDH).

#### Effect of providing rare tRNAs

Codon usage bias is a frequent problem encountered in the expression of eukaryotic proteins in *E. coli *[13]. Sequence analysis of *Cb*FDH revealed 13 especially rare triplets encoding arginine (agg, aga) and two rare isoleucine codons (ata) [14]. The pRSF-1b_FDH plasmid was therefore transformed into *E. coli *Rosetta 2 (DE3), a derivative of BL21 (DE3) that was specially designed to enhance recombinant production of eukaryotic proteins. Rosetta strains supply tRNAs for six codons that are rarely used in *E. coli *and include the above mentioned arginine and isoleucine codons. Using expression from pRSF-1b_FDH in *E. coli *Rosetta 2 (DE3), we could enhance the activity of *Cb*FDH by 11% as compared to the reference (Table 1).

#### Effect of induction conditions

It has been known that a decrease in induction temperature can generate positive effects on recombinant protein production, often resulting from a generally lowered expression rate or change in the relative rates of protein folding and aggregation [9]. We examined *Cb*FDH production in *E. coli *strains BL21 (DE3), BL21 star (DE3) and Rosetta 2 (DE3), using 18°C instead of 25°C during the induction phase. Table 1 shows that FDH-activities were raised between 12 and 38% by the application of a lower induction temperature. The highest *Cb*FDH activity of 614 U/g_CDW _was obtained by using a host strain with improved capability of handling rare codons and induction at 18°C (Table 1). Optimization of single-expression led to a 1.5-fold improvement in *Cb*FDH activity, equal to 28% of the total soluble protein in the *E. coli *extract.

### Co-expression of *Cb*FDH and *Ct*XR

#### Expression of two genes from pET-Duet vector

We have previously described the plasmid vector pETDuet_XR_FDH for co-expression of *Cb*FDH and *Ct*XR [3]. This vector was derived from a pET-Duet-1 plasmid and contained the gene of *Ct*XR and *Cb*FDH inserted in its first and second multiple cloning site (MCS1, MCS2), respectively. The order of placement of the two genes reflected the notion that the second inserted gene is generally expressed more strongly than the gene inserted first. MCS1 is missing a T7 terminator which leads to transcriptional read-through that destabilizes corresponding mRNAs [9,12]. Enzyme production was done in *E. coli *BL21 (DE3), and the XR activity of 1804 U/g_CDW _was comparable to the activity (U/g_CDW_) obtained under single gene expression conditions using a pET11a plasmid vector in *E. coli *BL21 (DE3) [15]. The FDH activity, by contrast, was just about one-fifth (85 U/g_CDW_) of the activity obtained under single gene expression conditions using pBTac1 in *E. coli *JM109 (Table 1, strain BL21_XR_FDH) [8].

Equal gene copy number and promoter strength provided by pET-Duet-1 led to imbalanced reductase and dehydrogenase activities. Expression of functional *Ct*XR in BL21_XR_FDH accounts for 30% of soluble protein in *E. coli*, 7.7-fold as compared to *Cb*FDH. To improve FDH activity under conditions of *Cb*FDH and *Ct*XR co-expression, we tried to repress *Ct*XR expression by gene copy number variations. Moreover, the host Rosetta 2 (DE3) assured provision of rare codon tRNAs and culture conditions found to be useful for FDH production under single gene expression conditions were applied.

#### Expression of two genes from two vectors

The pBR322-derived replicon of pET-Duet-1 determines a copy number of approximately 40 per cell [16]. The vector carries two T7*lac *promoters preceding MCS1 and MCS2 and an ampicillin resistance gene. Co-expression from multiple plasmids requires compatible replicons and different antibiotic resistances [16]. The newly constructed pRSF_FDH contains a RSF1030 replicon and a kanamycin resistance, both compatible with pET-Duet-1. An increase in *Cb*FDH gene copy number from ~ 40 to > 140 by co-transformation of BL21 (DE3) with pETDuet_XR_FDH and pRSF_FDH resulted in a two-fold increased FDH-activity from 85 to 146 U/g_CDW _(Table 1, strain BL21_XR_2FDH). Transformation of Rosetta 2 (DE3) harbouring the co-expression plasmid with pRSF_FDH resulted in 202 U/g_CDW_. A decrease in induction temperature from 25 to 18°C further improved *Cb*FDH activity in the cell free raw extract to 251 U/g_CDW _(Table 1, strain Rosetta_XR_2FDH). This is equal to a 3-fold increase in functional FDH expression with a concomitant *Ct*XR activity reduction from 1406 to 1140 U/g_CDW _(Table 2).

**Table 2:** Yields of *o*-chloroacetophenone reductions catalyzed by *E. coli *whole-cell biocatalysts variable in XR and FDH activities.

*E coli*	pH	XR (U/g_CDW_)	FDH (U/g_CDW_)	Substrate (mM)	Yield (mM)^1^	Yield (mM)^1, 2^
BL21_XR_FDH	7.5	1406	110	100	19	46

Rosetta_XR_2FDH	7.5	1140	251	100	35	67

BL21_XR_FDH	6.2	2226	48	100	27	78

Rosetta_XR_2FDH	6.2	2024	189	100	63	95

^1^Determined for a reaction time of 8 h. ^2^Additives: 36 μM Polymyxin B sulphate and 500 μM NAD^+^.

The obtained *Cb*FDH activity of 500 U/g_protein _is, to the best of our knowledge, the highest activity reported for co-expression strains so far [5,17]. Weckbecker et al. expressed *Cb*FDH and three further genes from compatible duet-plasmids. The alcohol dehydrogenase and *Cb*FDH were cloned into the first and second cloning site of the low copy number plasmid pACYCDuet-1. The host BL21 (DE3) was co-transformed with pETDuet-1 carrying the two subunits of the transhydrogenase. The obtained FDH activity of 210 U/g_protein _seems relatively high under induction conditions for several reasons [5]. First, the low copy number of the *Cb*FDH gene, second the co-expression of three further genes and third, the induction temperature of 37°C. However, the three co-expressed genes are of prokaryotic origin (*E. coli *transhydrogenase, *Lactobacillus kefir *alcohol dehydrogenase) whereas eukaryotic genes are co-expressed in the present study. The combined expression of *Cb*FDH and *Ct*XR in Rosetta 2 (DE3) adds up to 31% of soluble *E. coli *protein. Arg codons used in *Cb*FDH and *Ct*XR (aga, agg; [14]) count among the specifically rare codons in *E. coli *and are ~ 10-times more frequently used as compared to its natural occurrence in *E. coli*. *Cb*FDH carries furthermore a tandem rare Arg-codon double repeat (Arg210, Arg211; [14]). Ribosome stalling at the tandem rare codon double repeat might account for low *Cb*FDH expression at concurrent high *Ct*XR production [9]. *Cb*FDH co-expression with *Ct*XR requires a throttling in protein production velocity most probably owing to rare arg-codon dependence of both genes.

The high complexity of protein co-expression complicates the correlation of expression parameter to enzyme activity. We therefore performed a factorial design to figure out the most relevant variables influencing *Cb*FDH activity in co-expression strains. *Cb*FDH activities denoted in Table 1 served as response variable of a 2^3 ^factorial design using type of host, gene copy number and induction temperature as independent variables. All three factors turned out as significantly influencing *Cb*FDH activity by means of statistics. Gene copy numbers were most important in balancing *Cb*FDH and *Ct*XR co-expression. Exchange of BL21 by Rosetta 2 and a decrease in induction temperature from 25 to 18°C showed comparable effects on *Cb*FDH activity. Effects of interactions between factors are of minor importance to the *Cb*FDH activity. (Pareto chart of main effects and interactions between factors are shown in the Additional file 1.) Bioreductions of D-xylose and *o*-chloroacetophenone were used to study the effect of increased intracellular FDH-activity on initial rates and productivities.

### Bioreductions

*Ct*XR converts its natural substrate D-xylose and xenobiotic *o*-chloroacetophenone with specific activities of 12 and 4.4 U/mg, respectively. Sugar-bioreduction constitutes a completely different problem in whole cell biocatalysis as compared to reduction of aromatic ketones. The two main problems in whole cell biocatalysis - biocatalyst stability and substrate availability - are differentially affected by substrates with opposed polarities [3,4]. Whole cell reductions of hydrophilic xylose and hydrophobic *o*-chloroacetophenone were used to study the effect of increased FDH-activity under contrary conditions. The corresponding products, xylitol and (*S*)-1-(2-chlorophenyl)ethanol, are used in food-processing and pharmaceutical industry. The alternative food sweetener xylitol is produced in ton scale and chiral 1-(2-chlorophenyl)ethanols are key intermediates in the synthesis of a novel class of chemotherapeutic substances (PLK1 kinase inhibitors; [18-20]).

#### Xylitol production

**Figure 1:**
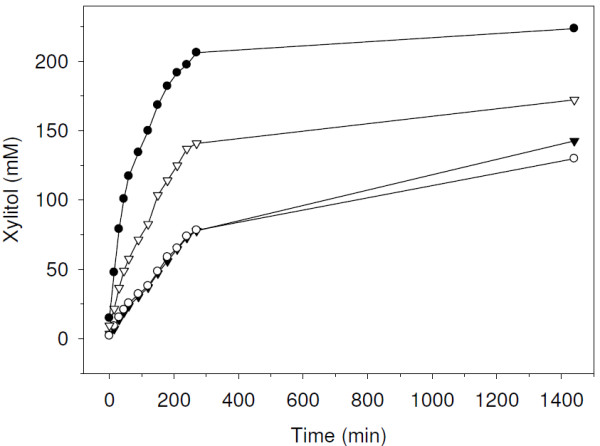
**Bioreductions of D-xylose catalyzed by whole cells and crude cell extracts of *E. coli *strains BL21_XR_FDH and Rosetta_XR_2FDH**. Type of catalyst is indicated by symbols: ● Rosetta_XR_2FDH crude extract; ○ Rosetta_XR_2FDH whole-cell catalyst; ∇ BL21_XR_FDH crude extract; ▼ BL21_XR_FDH whole-cell catalyst. Reaction conditions: D-xylose (250 mM), sodium formate (300 mM), cells (10 g_CDW_/L), 30°C.

We compared the strains BL21_XR_FDH and Rosetta_XR_2FDH in whole cell reductions of 250 mM D-xylose (Figure 1). Time courses of xylitol formation were, within the experimental error, identical for both strains. Initial rates (*r*_s_) of ~50 U/g_CDW _were calculated from the linear parts of time courses (0-30 min). After 24 h product yields of 55% with productivities of 2.0 g_product_/g_CDW _were obtained. We and others have previously shown that rates in whole cell biocatalysis are governed by the activity levels of enzymes and the substrate availability in the cell [3,4,21,22]. Similar xylose reduction rates for cells differing 2-fold in cofactor recycling activity suggest a main obstacle in substrate and/or co-substrate import into the cell. Kaup et al. used an *E. coli *whole cell catalyst co-expressing mannitol dehydrogenase and a bacterial FDH for the reduction of fructose. Mannitol yields were limited by the fructose uptake into the cell. Removal of mass transfer resistance by the co-expression of a fructose transporter led to a 14-fold increase in yields [21]. Protection of enzymes in the united cell structure is not required for the conversion of bio-compatible sugar substrates. We therefore repeated the bioreductions of xylose with disrupted cells (crude cell extracts) under otherwise identical conditions. We obtained *r*_s _for xylitol formation of 125 U/g_CDW _and 263 U/g_CDW _for BL21_XR_FDH and Rosetta_XR_2FDH, respectively (Figure 1). The 2.5 and 5-fold increases in *r*_s _obtained with the crude cell extracts as compared to whole cells identify substrate import into the cell as main limiting factor. Initial rates agree well with FDH-activities determined photometrically in the cell free crude extracts of BL21_XR_FDH (110 U/g_cdw_) and Rosetta_XR_2FDH (251 U/g_cdw_) (Table 1; induction at 18°C). Virtually identical *r*_s _values calculated from bioreductions and photometric FDH assays unmask the cofactor recycling rate as second limiting factor. The 2-fold higher initial rate and higher conversion obtained with the FDH optimized strain directly reflects the FDH-expression level in the cell which in turn rules out substrate or product inhibitions under reaction conditions. The combination of mass transfer relief and cofactor recycling improvement led to a conversion of > 90% with a productivity of 3.4 g_product_/g_CDW_.

#### *(*S*)-1-(2-chlorophenyl)ethanol production*

We compared *E. coli *BL21_XR_FDH and the FDH-optimized strain Rosetta_XR_2FDH in whole cell reductions of 100 mM *o*-chloroacetophenone. Product concentrations obtained with BL21_XR_FDH and Rosetta_XR_2FDH yielded 19 and 35 mM (*S*)-1-(2-chlorophenyl)ethanol, respectively (ee > 99.9; Table 2). Reduction of hydrophobic *o*-chloroacetophenone was therefore 1.8-fold improved by the 2.3-fold higher intracellular FDH activity of Rosetta_XR_2FDH (Table 2). Standard reactions and activity measurements were carried out in potassium phosphate buffer at pH 7.5; isolated xylose reductase however shows ~ 70% higher activities at pH 6.2. By lowering the reaction pH from 7.5 to 6.2 product yields increased 40% for the initial strain and 80% for the FDH optimized strain (Table 2). Higher reduction yields obtained with the FDH-optimized strain or at a more acidic reaction pH strongly indicate a permeabilizing effect of *o*-chloroacetophenone and its reduction product on *E. coli *cells. We have previously shown that the high toxicity of *o*-chloroacetophenone towards biocatalyts restricts the time of *E. coli *whole cell reductions to maximally ~5 h due to fast catalyst deactivation [4]. Mass transfer limitations on the one hand and substrate toxicity on the other hand requires cautious balancing of cell permeabilization. We used Polymyxin B sulphate to gently permeabilize the cell envelope and thereby further accelerate the uptake of ketone and formate. Reduction of mass transfer limitation over the cell membrane was reflected by increases in product concentrations to 78 and 95 mM obtained with BL21_XR_FDH and Rosetta_XR_2FDH, respectively. The enantiomeric excess of the product (*S*)-1-(2-chlorophenyl)ethanol was >99.9% in all experiments.

(*S*)-1-(2-chlorophenyl)ethanol formation was elevated by increasing intracellular FDH- or XR-activities. Product yield sensitivity for both enzyme activities suggests similar specific activities under process conditions. Reductase activities for *o*-chloroacetophenone are 2.7-fold reduced as compared to xylose activities in Table 2. Ketone reductase activity was determined at 10 mM substrate, which is the solubility limit of *o*-chloroacetophenone in phosphate buffer and roughly reflects the available substrate at 100 mM. Formate concentrations in FDH-activity assays and in whole cell reductions were ≥ 10-fold *K*_formate _(15 mM [8]) and saturating. The formate concentration in whole cell reductions was adjusted to 150 mM, 50 mM excess regarding the ketone concentration. Substrate availability seems to govern the specific activity of the whole cell catalyst not only in the case of xylose but also with *o*-chloroacetophenone. A limitation of substrate and co-substrate in the cell effects the activity of whole cell catalysts differentially, because XR-activity depends linearly on the ketone concentration (*K_o-chloroacetophenone _*≥ 10 mM [4]), whereas a formate concentration of 150 mM is 10-fold the *K*_formate_.

## Conclusions

A particular challenge in the development of 'designer bugs' based on *Cb*FDH is the low level of intracellular FDH in co-expression strains. Especially high overexpression of *Ct*XR restricted FDH co-expression to 85 U/g_CDW_. We used expression engineering to fine tune formate dehydrogenase and reductase activity levels in *E. coli*. Our strategy was to co-express *Ct*XR and *Cb*FDH based on the same inducible promoter for both genes but with different gene copy numbers. We furthermore used an *E. coli *host optimized for the expression of eukaryotic genes by supplying tRNAs for six codons that are rarely used in *E. coli*. Activities of *Ct*XR and *Cb*FDH were - after optimization of induction - 1140 U/g_CDW _and 251 U/g_CDW_, respectively. Improved FDH-activity resulted in full conversion of 250 mM D-xylose and 100 mM *o*-chloroacetophenone provided that mass transfer limitations are negligible. Full exploitation of higher FDH-activities required elimination of cell wall obstacle in the reduction of D-xylose. Hydrophobic *o*-chloroacetophenonen and (*S*)-1-(2-chlorophenyl)ethanol that destroy free *Ct*XR and *Cb*FDH within minutes [4], permeabilize the cell membrane and thereby facilitate substrate uptake. Further permeabilzation was done with the antibiotic Polymyxin B sulphate. The conversion of toxic, hydrophobic compounds by whole cells requires additional reaction engineering to protect the catalyst. A convenient method is the *in situ *extraction of hydrophobic compounds onto or into non-polar, second phases. Doig et al. (2002) have previously shown that the inhibitory effect of the lactone substrate bicycle[3.3.0]hept-2-en-6-one was overcome by a combination of substrate feeding and *in situ *substrate supply [23]. Strategies to overcome low cell activities and stabilities under process conditions comprise therefore expression engineering, elimination of mass transfer resistances and protection of the catalyst.

## Methods

### Chemicals

NADH (sodium salt; ≥98% pure), NAD^+ ^(free acid; ≥97.5% pure), D-xylose, and ampicillin were purchased at Roth (Karlsruhe, Germany). Sodium formate, Polymyxin B sulphate, kanamycin, chloramphenicol, and *o*-chloroacetophenone were obtained from Sigma-Aldrich (Vienna, Austria). B-Per^® ^Reagent was from Pierce (Rockford, IL, USA) and 1-(2-chlorophenyl)ethanol from Alfa Aesar (Karlsruhe, Germany). *Pfu *DNA polymerase was from Promega (Madison, WI, USA). dNTPs, T4 DNA ligase and restriction enzymes were obtained from MBI Fermentas (Flamborough, ON, Canada). Primers were synthesized by Invitrogen (Carlsbad, California, USA). Vectors pRSF-1b and pETDuet-1 were purchased at Novagen (VWR International GmbH, Vienna Austria). All other chemicals were from Sigma-Aldrich/Fluka or Roth, and were of the highest purity available.

### Strains and plasmids

The *E. coli *strains used were JM109 from Promega (Madison, WI, USA), BL21 (DE3) and Rosetta 2 (DE3) from Novagen (VWR International GmbH, Vienna, Austria) and BL21 star (DE3) from Invitrogen (Carlsbad, California, USA). All DNA manipulations and bacterial transformations were carried out according to standard protocols. The construction of the *E. coli *BL21 (DE3) (BL21_XR_FDH) harbouring *Ct*XR and *Cb*FDH genes on a pETDuet-1 vector (pETDuet_XR_FDH) was described elsewhere [3]. The *Cb*FDH gene was amplified from pETDuet_XR_FDH by a PCR using *Pfu *DNA polymerase and primers providing *Pag*I (compatible ends to *Nco*I) and *Avr*II restriction sites.

Forward primer: 5'- GGTGGTTCATGAAGATCGTTTTAG- 3'

Reverse primer: 5'- GTAAACACGATAAGAAATAACCTAGGGGTGGT- 3'

The *Cb*FDH gene was cloned into the multiple cloning site of pRSF-1b (*Nco*I, *Avr*II) prior to verification of correct integration by sequencing (pRSF_FDH). Bacterial transformation followed a standard electroporation protocol. For single-expression of *Cb*FDH, pRSF_FDH was transformed into BL21 (DE3), BL21 (DE3) star and Rosetta 2 (DE3), for co-expression these strains were co-transformed with pETDuet_XR_FDH. Constructs are summarized in Table 3.

**Table 3:** *E. coli *expression strains addressed in this study

*E. coli host*	Plasmid	Enzyme	Antibiotic	Abbreviation
JM109	pBTac1	*Cb*FDH	Amp^1^	JM109_FDH [8]

BL21 (DE3)	pRSF-1b	*Cb*FDH	Kan^2^	BL21_FDH

Rosetta 2 (DE3)	pRSF-1b	*Cb*FDH	Cam^3^, Kan	Rosetta_FDH

BL21 star (DE3)	pRSF-1b	*Cb*FDH	Kan	Star_FDH

BL21 (DE3)	pETDuet-1	*Ct*XR, *Cb*FDH	Amp	BL21_XR_FDH

BL21 (DE3)	pETDuet-1 pRSF-1b	*Ct*XR, *Cb*FDH; *Cb*FDH	Amp, Kan	BL21_XR_2FDH

Rosetta 2 (DE3)	pETDuet-1	*Ct*XR, *Cb*FDH	Cam, Amp	Rosetta_XR_FDH

Rosetta 2 (DE3)	pETDuet-1 pRSF-1b	*Ct*XR, *Cb*FDH; *Cb*FDH	Cam, Amp, Kan	Rosetta_XR_2FDH

BL21 star (DE3)	pETDuet-1 pRSF-1b	*Ct*XR, *Cb*FDH; *Cb*FDH	Amp, Kan	Star_XR_2FDH

^1 ^Ampicillin 115 mg/L; ^2 ^kanamycin 50 mg/L; ^3 ^chloramphenicol 34 mg/L.

### Cultivation of strains

*E. coli *strains were grown in 1000 mL baffled shaken flasks containing 200 mL of LB media supplemented with antibiotics according to Table 3. Flasks were shaken at 130 rpm and 37°C in a Certomat^® ^BS-1 incubator from Sartorius. At an optical density of 1.1 (± 10%) cultures were cooled to 25 or 18°C and protein production was induced by addition of 250 μM isopropyl-β-D-thiogalactopyranosid (IPTG). After 20 h of cultivation, cells were harvested by centrifugation. Samples were taken and the B-Per^® ^cell lysis reagent was used for protein extraction prior to enzyme activity measurements.

### Enzyme activity measurements in the cell-free extract

Reductase and dehydrogenase activities were assayed spectrophotometrically at 340 nm by monitoring the reduction or oxidation of NAD(H) over a time period of 5 minutes (rates of 0.05 - 0.10 ΔA/min). One unit of enzyme activity refers to 1 μmol of NAD(H) consumed or formed per minute. All activity measurements were performed with a Beckman DU-800 spectrophotometer thermostated at 25°C. Xylose reductase activity was either determined with the native substrate D-xylose (700 mM) or with *o*-chloroacetophenone (10 mM). Reactions were started with addition of NADH in a final concentration of 310 μM. The assay for formate dehydrogenase activity contained 200 mM sodium formate and the reaction started with the addition of 2 mM NAD^+^. Activity assays were performed in 100 mM potassium phosphate buffer, pH 7.5 or 6.2. Five % ethanol was added to the buffer to enhance the solubility of *o*-chloroacetophenone. Measured rates were corrected for the appropriate blank readings accounting for non-specific oxidation or reduction of NAD(P)(H) by the cell extracts.

### Whole-cell bioreductions

Experiments were carried out at 30°C on an end-over-end rotator (SB3 from Stuart) at 30 rpm.

#### D-Xylose

Biomass of each strain was divided into halves. One half of the cell material was diluted to 10 g_CDW_/L with 100 mM potassium phosphate buffer pH 7.5. 10 mL of the cell suspension were filled into a 15 mL Sarstedt tube with 300 mM co-substrate sodium formate added. The second half of the cells were disrupted by three passages through a French press (American Instrument Company, Silver Springs, Maryland, USA) operated at an applied pressure of 1000 psi. The crude cell extracts obtained were diluted with 100 mM potassium phosphate buffer, pH 7.5 to a concentration equivalent to 10 g_CDW_/L. After ultra-centrifugation, 10 mL of supernatant was transferred into a 15 mL Sarstedt tube and supplemented with 300 mM sodium formate and 500 μM NAD^+^. Conversions were started by the addition of 250 mM xylose. 200 μL samples were taken over time and the reaction was stopped after 24 h by the addition of ethanol in a 1:1 (v/v) ratio. Centrifugation separated the biomass and the product-containing supernatant was prepared for high performance liquid chromatography (HPLC).

#### o-Chloroacetophenone

*E. coli *cells were diluted to a concentration of 40 g_CDW_/L with 100 mM potassium phosphate buffer of either pH 7.5 or 6.2. The co-substrate sodium formate was added in a concentration of 150 mM, 50 mM excess as compared to the substrate *o*-chloroacetophenone. Reactions were started by the addition of *o*-chloroacetophenone which was pre-dissolved in ethanol to enhance its solubility. Final ethanol concentrations were 5%. Polymyxin B sulphate and NAD^+ ^were added in concentrations of 36 and 500 μM, respectively, to the reaction mix. Total reaction volumes were 1 mL and bioreductions of *o*-chloroacetophenone were performed in 2 mL Eppendorf tubes. After eight hours reactions were stopped by the addition of ethanol in a 1:1 (v/v) ratio. Cells were then separated by centrifugation and the supernatant was analyzed by chiral HPLC.

### Analytical methods

HPLC-analysis of whole-cell reductions was performed on a LaChrom HPLC system (Merck-Hitachi) equipped with an L-7400 UV-detector (210 nm), a Merck L-7490 RI detector, and a thermo-stated column oven.

#### D-Xylose

Samples were analysed on an Aminex HPX-87H column (Bio Rad Laboratories, Vienna, Austria) using sulphuric acid (5 mM H_2_SO_4_) as eluent at a flow rate of 0.6 mL/min and a temperature of 65°C. For xylitol peak identification and quantification authentic standards with known concentrations were measured. Reported yields of xylitol product are always from analytical data, product isolation was beyond the scope of this study.

#### o-Chloroacetophenone

Samples were analysed on a reversed phase CHIRALPAK AD-RH column from Daicel (VWR International, Vienna, Austria) using an acetonitrile-water mixture (20:80 v/v) at a flow rate of 0.5 mL/min and a column temperature of 40°C. For alcohol peak identification standards with known concentrations were measured. Reported product yields are always from analytical data.

## Competing interests

The authors declare that they have no competing interests.

## Authors' contributions

KM and KS performed the experiments and analyzed the experimental data. KM was involved in experimental design and manuscript preparation. BN has made substantial contributions to conception, interpretation of data and revised the manuscript. RK designed the experiments, interpreted the results and drafted the manuscript. All authors read and approved the final manuscript.

## Supplementary Material

Additional file 1 **Factorial design**. *Cb*FDH activity measured in the cell-free extract was specified as response variable of a 2^3 ^factorial design. Type of host (A), number of plasmids (B) and induction temperature (C) were chosen as experimental factors. Click here for file
