# Supplementary material for: Host cell and expression engineering for development of an E. coli ketoreductase catalyst: Enhancement of formate dehydrogenase activity for regeneration of NADH
Source: Microb Cell Fact. 2012 Jan 11;11:7. doi: 10.1186/1475-2859-11-7 (PMC3278346; doi:10.1186/1475-2859-11-7)
Supplement: Additional file 1 — Factorial design. CbFDH activity measured in the cell-free extract was specified as response variable of a 23 factorial design. Type of host (A), number of plasmids (B) and induction temperature (C) were chosen as experimental factors. [file 1475-2859-11-7-S1.DOC]

# Host cell and expression engineering for development of an *E. coli* ketoreductase catalyst: Enhancement of formate dehydrogenase activity for regeneration of NADH

Katharina Mädje^1^, Katharina Schmölzer^1^, Bernd Nidetzky*^,1^, Regina Kratzer*^,1^

^1^Institute of Biotechnology and Biochemical Engineering, Graz University of Technology (TUG), Petersgasse 12/1, A-8010 Graz, Austria

Email addresses:

Katharina Mädje - [maedje@student.tugraz.at](mailto:maedje@student.tugraz.at)

Katharina Schmölzer - [katharina.schmoelzer@acib.at](https://webmail.tugraz.at/horde/imp/message.php?mailbox=INBOX&index=1639)

Bernd Nidetzky - [bernd.nidetzky@tugraz.at](mailto:bernd.nidetzky@tugraz.at)

Regina Kratzer - [regina.kratzer@tugraz.at](mailto:regina.kratzer@tugraz.at)

*Corresponding authors

**Additional file 1**

**Factorial design**

Table A1

Figure A1

**Factorial design**

*Cb*FDH activity measured in the cell-free extract was specified as response variable of a 2^3^ factorial design. Type of host (A), number of plasmids (B) and induction temperature (C) were chosen as experimental factors.

Response was modelled by the following polynomic equation: *R = aA + bB + cC + dAB + eAC + fBC + g* (STATGRAPHICS Centurion XVI, Version 16.1.11).

Table A1: *Cb*FDH activity as a function of host, number of plasmids and induction temperature. Normalized values are used.

| **A**  ***E coli* strain**  BL21 (DE3) = -1  Rosetta2 (DE3) = +1 | **B**  **Number of plasmids**  pETDuet-1 = -1  pETDuet-1 & pRSF = +1 | **C**  **Induction temperature**  18°C = -1  25°C = +1 | ***Cb*FDH activity**  (U/g_CDW_) |
| --- | --- | --- | --- |
| -1 | -1 | -1 | 110 |
| -1 | +1 | -1 | 186 |
| -1 | +1 | +1 | 146 |
| -1 | -1 | +1 | 85 |
| +1 | -1 | -1 | 138 |
| +1 | +1 | -1 | 251 |
| +1 | +1 | +1 | 202 |
| +1 | -1 | +1 | 100 |

Coefficient estimates of effects and interactions are shown as Pareto chart in Figure A1. All 3 factors appear to have a significant effect on *Cb*FDH activity, since bars for each factor extends beyond the 5% significance vertical line. Interactions are of minor importance.


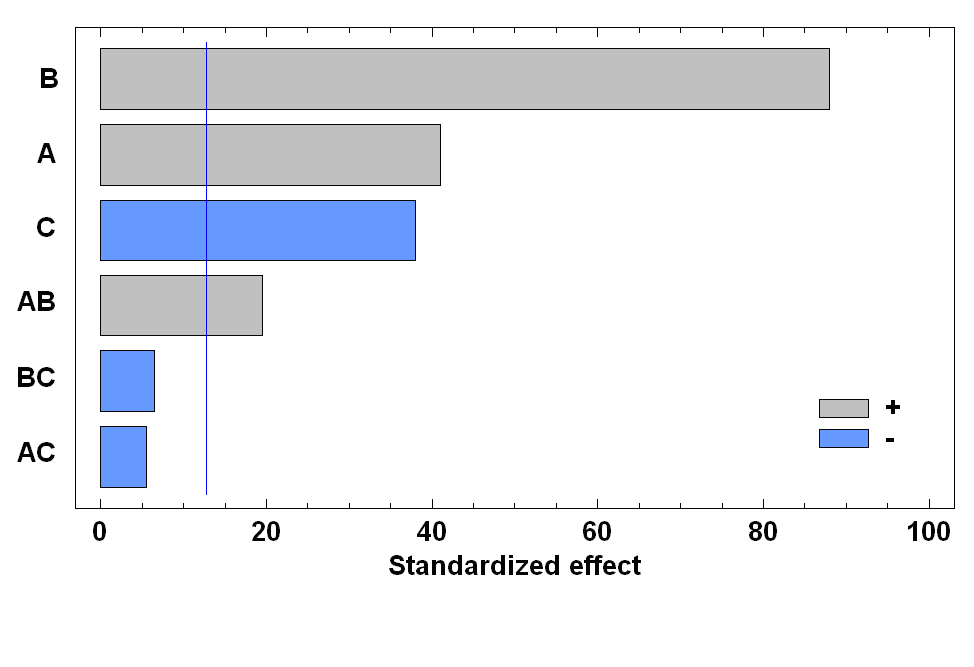


Figure A1: Standardized Pareto chart for gene copy number, host and induction temperature effects on *Cb*FDH activity.
